# Supplementary material for: Western-Type Helicobacter pylori CagA are the Most Frequent Type in Mongolian Patients
Source: Cancers (Basel). 2019 May 24;11(5):725. doi: 10.3390/cancers11050725 (PMC6562502; doi:10.3390/cancers11050725)
Supplement: Supplementary file 1 [file cancers-11-00725-s001.pdf]

Supplementary Material: Western-Type *Helicobacter pylori* CagA are the Most Frequent Type in Mongolian Patients

Tegshee Tserentogtokh, Boldbaatar Gantuya, Phawinee Subsomwong, Khasag Oyuntsetseg, Dashdorj Bolor, Yansan Erdene-Ochir, Dashdorj Azzaya, Duger Davaadorj, Tomohisa Uchida, Takeshi Matsuhisa and Yoshio Yamaoka

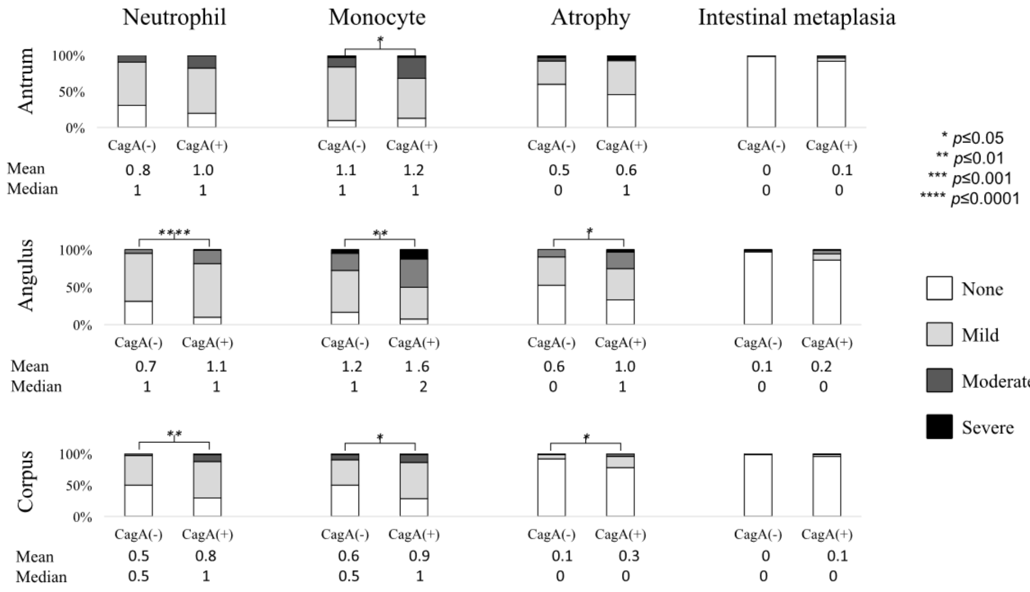

Figure S1. CagA status based on histological status. The distribution and mean values of histological status are shown based on *cagA*-negative and *cagA*-positive cases.

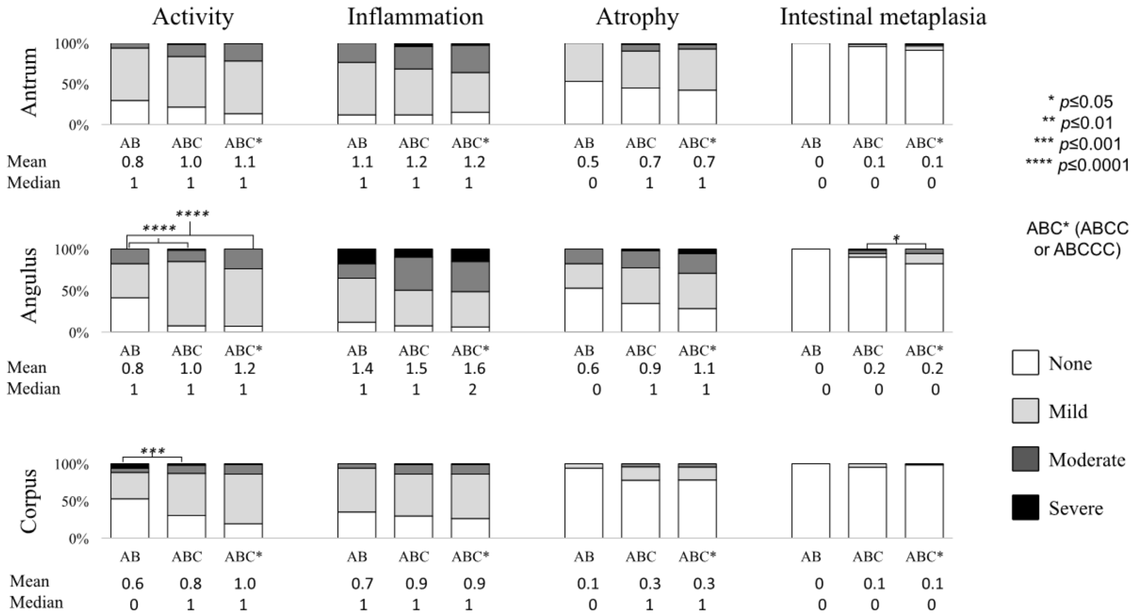

Figure S2. Western-type CagA types based on histological status. The distribution and mean values of histological status are shown based on Western-type CagA types.

Table S1. Patient demography.

Disease status	Mean ages	Ages (Std. Deviation)	Minimum age	Maximum age	Female patients	Male patients
**Gastric cancer**	59.9	14.4	36	81	5	12
**Gastritis**	42.3	13.6	16	79	246	95
**Ulcers**	46.6	9.2	30	58	7	3

Table S2. The CagA and *vacA* genotyping based on geography.

	cagA genotypes		Ulaanbaatar *n* = 117 (%)	Uvs *n* = 21 (%)	Khuvsgul *n* = 31 (%)	Khentii *N* = 89 (%)	Umnugovi *n* = 93 (%)
**CagA**	**Negative**	**(-)**	24 (20.5)	7 (33.3)	5 (16.1)	16 (18)	10 (10.8)
	**Western**	**AB**	8 (6.8)	0	3 (9.7)	2 (2.2)	2 (2.2)
		**ABB**	1 (0.8)	0	0	0	0
		**ABBB**	0	0	0	0	1 (1.1)
		**ABBC**	0	0	0	1 (1.1)	1 (1.1)
		**ABC**	47 (40.5)	5 (23.8)	12 (38.7)	31 (34.8)	48 (51.6)
		**ABCC**	34 (29)	9 (42.9)	10 (32.3)	35 (39.3)	21 (22.6)
		**ABCCC**	0 (0)	0	1 (3.2)	2 (2.2)	2 (2.2)
	**East-Asian**	**ABD**	1 (0.8)	0	0	3 (2.2)	6 (6.5)
		**ABBD**	1 (0.8)	0	0	0	2 (2.2)
	**Hybrid**	**ABDC**	1 (0.8)	0	0	0	0
vacA	**S region**	**s1**	96 (82)	13 (65)	24 (82.8)	71 (81.6)	80 (88.9)
		**s2**	21 (18)	7 (35)	5 (17.2)	16 (18.4)	10 (11.1)
	**M region**	**m1**	64 (54.7)	11 (55)	21 (72.4)	44 (50.6)	50 (55.6)
		**m2**	53 (45.3)	9 (45)	8 (27.6)	43 (49.4)	40 (44.4)

Table S3. Multivariate analysis for CagA types based on histological features of gastritis

Histology diagnosis	CagA types	P value	OR	Lower 95% C.I.	Upper 95% C.I.
Neutrophil infiltration	Negative*				
	AB	NS	0.9	0.2	4
	ABC	0.002	5.8	1.9	18
	ABCC	0.003	10.8	2.3	51
	ABCCC	NS	0.9	0.1	9
	ABD	NS	2.1	0.2	18
Monocyte infiltration	Negative*				
	AB	NS	0.5	0.05	6
	ABC	NS	1.0	0.2	5
	ABCC	NS	3.8	0.3	42
	ABCCC	-	-	-	-
	ABD	-	-	-	-
Atrophy	Negative*				
	AB	NS	0.8	0.2	2
	ABC	NS	1.7	0.8	3
	ABCC	0.04	2.2	1.0	5
	ABCCC	NS	0.8	0.1	5
	ABD	NS	2.0	0.4	10
Intestinal metaplasia	Negative*				
	AB	-	-	-	-
	ABC	0.02	4.5	1.3	15
	ABCC	0.002	7.0	2.0	24
	ABCCC	0.03	9.8	1.3	77
	ABD	0.002	14.0	2.7	72

*used as control, NS (not significant)

Table S4. Multivariate analysis for *vacA* genotypes based on histological features of gastritis.

Histology diagnosis	*vacA* genotypes	*p* value	OR	Lower 95% C.I.	Upper 95% C.I.
Neutrophil infiltration	s2/m2*			-	
	s1/m2	0.02	3.7	1.2	11.5
	s1/m1	0.001	5.4	2.0	14.9
Monocyte infiltration	s2/m2*			-	
	s1/m2	NS	0.6	0.1	3.4
	s1/m1	NS	3.4	0.5	24.4
Atrophy	s2/m2*			-	
	s1/m2	NS	1.4	0.7	2.9
	s1/m1	0.04	2.0	1.0	3.8
Intestinal metaplasia	s2/m2*			-	
	s1/m2	0.04	3.9	1.1	14.0
	s1/m1	0.003	6.2	1.9	20.7

*used as control, NS (not significant)
